# Phase Ib/II Study of the Efficacy and Safety of Binimetinib (MEK162) Plus Panitumumab for Mutant or Wild-Type *RAS* Metastatic Colorectal Cancer

**DOI:** 10.1093/oncolo/oyad210

**Published:** 2023-08-19

**Authors:** Eric Van Cutsem, Rona Yaeger, Jean-Pierre Delord, Josep Tabernero, Lillian L Siu, Michel Ducreux, Salvatore Siena, Elena Elez, Stefan Kasper, Thomas Zander, Neeltje Steeghs, Danielle Murphy, Michelle Edwards, Zev A Wainberg

**Affiliations:** Digestive Oncology, University Hospitals Gasthuisberg, Leuven and KU Leuven, Leuven, Belgium; Department of Medicine, Memorial Sloan Kettering Cancer Center, New York, NY, USA; Medical Oncology Department, Institut Claudius Regaud, IUCT-Oncopole, Toulouse, France; Medical Oncology Department, Vall d’Hebron Campus Hospital and Institute of Oncology (VHIO), IOB-Quiron, UVic-UICC, Barcelona, Spain; Division of Medical Oncology and Hematology, Princess Margaret Cancer Centre, University Health Network, Toronto, ON, Canada; Department of Medical Oncology, Gustave Roussy, Cancer Campus, Université Paris-Saclay, Villejuif, France; Department of Oncology and Hemato-Oncology, Università degli Studi di Milano, Milan, Italy; Department of Oncology, Niguarda Cancer Center, Grande Ospedale Metropolitano Niguarda, Milan, Italy; Medical Oncology Department, Vall d’Hebron Campus Hospital and Institute of Oncology (VHIO), Universitat Autònoma de Barcelona (UAB), Barcelona, Spain; West German Cancer Center, Department of Medical Oncology, University Hospital Essen, Essen, Germany; Department of Internal Medicine, Center for Integrated Oncology Aachen-Bonn-Cologne, University of Cologne, Duesseldorf, Germany; Department of Internal Medicine I, Gastrointestinal Cancer Group Cologne (GCGC), University Clinic Cologne, Cologne, Germany; Department of Medical Oncology and Clinical Pharmacology, Netherlands Cancer Institute, Amsterdam, The Netherlands; Translational Oncology, Pfizer, La Jolla, CA, USA; North American Medical Affairs, Pfizer, New York, NY, USA; Division of Hematology/Oncology, David Geffen School of Medicine at UCLA, Los Angeles, CA, USA

**Keywords:** binimetinib, panitumumab, colorectal cancer, *RAS* mutation, *RAS* wild type

## Abstract

**Introduction:**

Activating *RAS* gene mutations occur in approximately 55% of patients with metastatic colorectal cancer (mCRC) and are associated with poorer clinical outcomes due to epidermal growth factor receptor (EGFR) blockade resistance. Combined EGFR and mitogen-activated protein kinase (MEK) inhibition may extend response to EGFR inhibition and overcome acquired resistance. This phase Ib/II dose escalation trial evaluated the safety and activity of dual inhibition with binimetinib (MEK1/2 inhibitor) and panitumumab (EGFR inhibitor [EGFRi]) in patients with *RAS* mutant or *BRAF* wild type (WT)/*RAS* WT mCRC.

**Methods:**

Phase Ib dose escalation started with binimetinib 45 mg twice daily plus panitumumab 6 mg/kg administered every 2 weeks. In the phase II study, patients with measurable mCRC were enrolled into 4 groups based on previous anti-EGFR monoclonal antibody therapy and *RAS* mutational status.

**Results:**

No patients in the phase Ib portion (*n* = 10) had a response; 70% of patients had stable disease. In the phase II portion (*n* = 43), overall response rate (ORR, confirmed) was 2.3% with one partial response in the *RAS* WT group, DCR was 30.2%, and median progression-free survival was 1.8 months (95%CI, 1.6-3.3). All patients experienced ≥1 adverse event, with the most common being diarrhea (71.7%), vomiting (52.8%), nausea (50.9%), fatigue (49.1%), dermatitis acneiform (43.4%), and rash (41.5%). Most patients required treatment interruption or dose reduction due to difficulties tolerating treatment.

**Conclusions:**

The combination of binimetinib and panitumumab had substantial toxicity and limited clinical activity for patients with mutant or WT *RAS* mCRC, independent of EGFRi treatment history (Trial registration: NCT01927341).

## Teaser

This article reports the results of a study that evaluated the combination of the MEK1/2 inhibitor binimetinib and the EGFR inhibitor panitumumab in patients with *RAS* -mutant or *BRAF* wild type/*RAS* wild-type metastatic colorectal cancer.

**Implications for Practice**

Epidermal growth factor receptor (EGFR) protein and the MAPK pathway (including the proteins RAS, RAF, MEK1/2, and ERK) are involved in cell growth and survival; activation of any of these proteins can cause cells to divide more rapidly, allowing cancer to develop. People with metastatic colorectal cancer (mCRC) tumors with activated RAS are resistant to anticancer medicines targeting EGFR due to activation of the MAPK pathway, limiting treatment options. Therefore, we used a combination of medicines. We tested panitumumab, which blocks EGFR, with binimetinib, which blocks MEK1/2, to see if resistance could be overcome. Adding binimetinib to panitumumab did not improve outcomes for people with mCRC with activated or nonactivated RAS regardless of a person’s previous history with EGFR blockers. In addition, many people found this combination of medicines hard to tolerate. This work showed that binimetinib plus panitumumab has limited benefit for people with activated or nonactivated RAS mCRC.

## Introduction

Monoclonal antibodies targeting epidermal growth factor receptors (EGFRs) have led to significant improvements in survival for patients with metastatic colorectal cancer (mCRC).^1,2^ Patients with *RAS* wild-type (WT) mCRC, whose primary tumor is located on the left side of the colon, can respond to treatment with EGFR inhibitors (EGFRi), such as cetuximab and panitumumab,^3,4^ but acquired resistance inevitably develops.^3,5^ Activating mutations in the *RAS* genes (*KRAS* and *NRAS*) are relatively common and occur in approximately 55% of patients with mCRC.^6^ Such mutations are associated with poorer clinical outcomes compared with patients with *RAS* WT tumors, and they confer resistance to EGFR blockade, further limiting treatment options.^2,4,6^

Studies of acquired resistance to EGFRi show that progressing tumors remain dependent on extracellular signal-regulated kinase (ERK) activation but that resistance alterations bypass EGFR inhibition to maintain ERK signaling.^7-9^ The most common resistance alterations identified in patient samples consist of *RAS* mutations, which lead to independent activation of ERK signaling downstream of EGFR or EGFR ectodomain mutations, which interfere with antibody binding.^7,10-12^ Preclinical data suggest that combined inhibition of EGFR and mitogen-activated protein kinase (MEK) may be able to extend response to EGFR inhibition and overcome acquired resistance.^8,9,11^ In mCRC, *RAS* mutations confer constitutive activation of the RAS-RAF-MEK-ERK signaling pathway.^6^ EGFR may be feedback suppressed by ERK activation in *RAS* mutant tumors.^13^ MEK is a key node in the MAPK pathway downstream of RAS, and preclinical models suggest MEK dependence of *RAS*-mutant mCRC. However, clinical trials with single-agent MEK inhibitors (MEKi) have shown limited activity in *RAS-*mutant (*RAS* MUT) mCRC,^14^ potentially due to the release of EGFR from feedback inhibition. Together, these data provided the rationale for this trial, which studied combined MEK and EGFR inhibition in patients with *RAS* WT, EGFRi-naïve or pretreated, or *RAS* MUT mCRC, including patients with secondary *RAS* mutations after prior EGFRi.

Binimetinib (MEK162) is a selective, potent allosteric inhibitor of MEK1/2 that has demonstrated antitumor activity in preclinical models.^15-17^ Binimetinib has been shown to markedly inhibit ERK phosphorylation in human cell lines and preferentially inhibits the proliferation of cells harboring *RAS* mutations.^16^ Furthermore, in the recent BEACON phase III study (NCT02928224), binimetinib demonstrated antitumor activity when coadministered to patients with *BRAF* V600E-mutant mCRC.^18^

This trial evaluates the combination of binimetinib and the EGFR inhibitor panitumumab in patients with *RAS* MUT or *BRAF* WT/*RAS* WT mCRC.

## Materials and Methods

### Study Design

This was a 2-part, open-label, multicenter study (NCT01927341) comprising a phase Ib dose escalation followed by a phase II clinical efficacy evaluation and safety assessment of the combination treatment binimetinib + panitumumab. For phase II, 4 patient populations were enrolled based on previous anti-EGFR monoclonal antibody (mAb) therapy and *RAS* mutational status.

The study was conducted at 8 sites in the US, Belgium, Canada, France, Germany, Italy, Netherlands, and Spain. The study protocol was approved by the independent ethics committee or institutional review board at each site, and the study was conducted according to International Council for Harmonisation of Technical Requirements for Registration of Pharmaceuticals for Human Use guidelines concerning Good Clinical Practice. Written informed consent was obtained from all patients before screening.

### Study Population

Both phases of the study enrolled adult patients (aged ≥18 years) with histologically or cytologically confirmed mCRC, evidence of measurable disease per Response Evaluation Criteria in Solid Tumors version 1.1 (RECIST 1.1), Eastern Cooperative Oncology Group performance status of 0 to 2, and written documentation of either *RAS* WT or somatic mutations in exon 2 (codons 12/13), 3 (codons 59/61), or 4 (codons 117/146) in *KRAS* or *NRAS* in their medical history.

For phase Ib, eligible patients had mCRC that had progressed on or following standard therapy or were determined to be patients for whom no standard therapy existed. For phase II, patients were eligible based on their previous anti-EGFR mAb therapy and *RAS* mutational status, resulting in 4 subgroups: (1) *RAS* MUT, EGFRi-naïve, (2) acquired *RAS* MUT, EGFRi pretreated, (3) *RAS* WT, EGFRi pretreated, and (4) *RAS* WT, EGFRi-naïve. Patients in the “acquired *RAS* MUT, EGFRi pretreated” group were diagnosed with an acquired *RAS* mutation, with either a new biopsy specimen upon study entry or from archival tissue collected after last therapy treatment. Patients in the pretreated subgroups had previously received anti-EGFR mAbs but not tyrosine kinase inhibitor therapy. All patients in phase II had experienced progression on or intolerance to ≥2 prior fluoropyrimidine-containing chemotherapy regimens, including irinotecan and oxaliplatin for metastatic disease, and had no remaining standard therapy options according to the investigator’s assessment.

### Study Objectives and Endpoints

The objective of phase Ib was to determine the maximum tolerated dose (MTD) of binimetinib + panitumumab. The MTD is defined as the highest combination drug dosage not causing medically unacceptable dose-limiting toxicities (DLTs) in more than 35% of treated patients in the first cycle of treatment, based on an adaptive 5-parameter Bayesian logistic regression model. DLTs were defined as adverse events (AEs) or clinically significant abnormal laboratory values assessed as unrelated to disease, disease progression, intercurrent illness, or concomitant medications that occurred within the first 28 days of treatment and met prespecified criteria. Infusion-related reactions were not considered DLTs.

The primary endpoint in phase Ib was the incidence of DLTs in the first 28-day cycle of treatment (cycle 1). Secondary endpoints included frequency and severity of AEs, overall response rate (ORR) per RECIST 1.1, progression-free survival (PFS), duration of response (DOR), and disease control rate (DCR). In phase II, the primary endpoint was ORR, and secondary endpoints included frequency and severity of AEs, PFS, DOR, and DCR.

AEs of special interest (AESI) were those for which there was a clinical interest related to the mechanism of action of the drug under investigation. These AESIs were defined on the basis of signals observed from previous studies in the binimetinib clinical development program and/or known class effects of other MEKi.

### Study Treatment and Procedures

In phase Ib, patients received a starting dose of binimetinib 45 mg twice daily (BID) + panitumumab 6 mg/kg once every 2 weeks (Q2W), the US Food and Drug Administration approved doses of both agents at the time. Dose de-escalation was planned as needed until the MTD/recommended phase II dose (RP2D) was reached. In phase II, patients received the MTD/RP2D of binimetinib + panitumumab, as determined during phase Ib.

Tumor response was evaluated locally by the investigator according to RECIST 1.1, using computed tomography scans and/or magnetic resonance imaging with intravenous contrast. Scans were performed at screening/baseline and then every 8 weeks (two 28-day cycles) until disease progression. Safety was assessed throughout the study, and AEs were coded using Medical Dictionary for Regulatory Activities version 18.1 terminology. Toxicity was assessed according to the Common Terminology Criteria for Adverse Events version 4.03. Per protocol, antidiarrheal medication was recommended at the first sign of abdominal cramping, loose stools, or overt diarrhea; skin toxicity prophylaxis was to be initiated 24 hours prior to first treatment with binimetinib or later as needed. Patients continued treatment until disease progression, development of unacceptable toxicity, or withdrawal of informed consent.

### Statistical Analyses

The MTD/RP2D of the combination treatment was estimated based on the anticipated probability of DLTs in cycle 1 for patients in the dose-determining set, which consisted of all phase Ib patients who met specified minimum exposure criteria and had sufficient safety evaluations during cycle 1 or who discontinued earlier due to a DLT during cycle 1.

Efficacy analyses included all patients who received any dose of binimetinib or panitumumab. An estimation approach was to be used to assess exploratory efficacy for patient subgroups *RAS* MUT, EGFRi-naïve; acquired *RAS* MUT, EGFRi pretreated; and *RAS* WT, EGFRi pretreated with 15 patients to be recruited for each subgroup. For patients in the *RAS* WT, EGFRi-naïve subgroup, given a historical response rate of 17% for patients receiving single agent panitumumab, an observed ORR greater than 28% was considered a clinically significant improvement, and the sample size was set to 35 patients. The ORR was defined as the proportion of patients with a best overall response of confirmed complete response (CR) or confirmed partial response (PR) per RECIST 1.1. The ORR was summarized with exact binomial 95% confidence intervals (CIs), generated according to the Clopper-Pearson method. PFS was defined as the time from the start of treatment to progression (first documented progression) or death from any cause. Overall survival (OS) was defined as the time from the start of treatment to the date of death from any cause. If the patient was not known to have died, survival was censored at the date of last contact when the patient was known to be alive. Kaplan-Meier analyses of PFS and OS were performed. The DCR was defined as the proportion of patients with a best overall response of confirmed CR, confirmed PR, or stable disease.

The safety analysis set included all patients who received ≥1 dose of binimetinib or panitumumab and had ≥1 valid postbaseline safety assessment.

## Results

### Patient Demographics and Disease Characteristics

**Figure 1.**
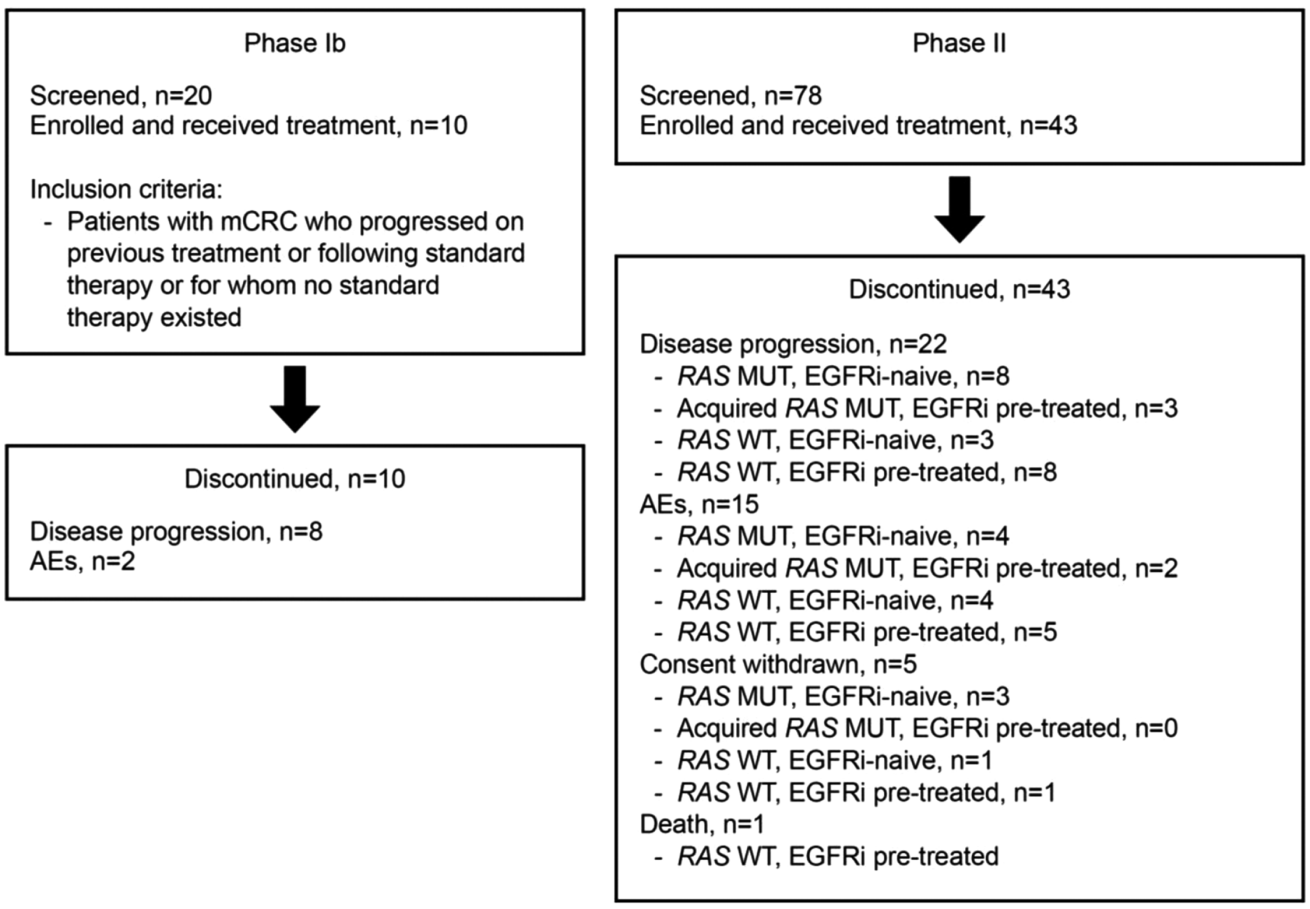
Patient flow diagram. Abbreviations: AE: adverse event; EGFRi: epidermal growth factor receptor inhibitor; mCRC: metastatic colorectal cancer; MUT: mutation; *RAS*: rat sarcoma viral oncogene homolog; WT: wild type.

In total, 53 patients were enrolled between November 19, 2013 and January 25, 2016: 10 in phase Ib and 43 in phase II (database lock: August 11, 2016; Fig. 1). In phase II, patients were grouped according to previous treatment and *RAS* mutational status: *RAS* MUT, EGFRi-naïve (*n* = 15); acquired *RAS* MUT, EGFRi pretreated (*n* = 5); *RAS* WT, EGFRi pretreated (*n* = 15); and *RAS* WT, EGFRi-naïve (*n* = 8).

Overall, the median age at enrollment was 55.0 (range 30-79) years, and the majority of patients were White (85%; Table 1). Patient demographics and baseline disease characteristics were generally similar across treatment groups in phase II (Table 1).

**Table 1.** Patient demographics and baseline characteristics.

	Phase Ib (*n* = 10)	Phase II					All patients (*N* = 53)
		*RAS* MUT, EGFRi-naïve (*n* = 15)	Acquired *RAS* MUT, EGFRi pretreated(*n* = 5)	*RAS* WT, EGFRi-naïve (*n* = 8)	*RAS* WT, EGFRi pretreated (*n* = 15)	All phase II (*n* = 43)	
**Sex**							
** Female**	4 (40.0)	9 (60.0)	4 (80.0)	5 (62.5)	9 (60.0)	27 (62.8)	31 (58.5)
** Male**	6 (60.0)	6 (40.0)	1 (20.0)	3 (37.5)	6 (40.0)	16 (37.2)	22 (41.5)
**Age, years**							
** Median (range)**	54 (38-70)	56 (36-71)	55 (49-75)	55 (39-69)	58 (30-79)	55 (30-79)	55 (30-79)
** ≥65**	2 (20.0)	2 (13.3)	2 (40.0)	2 (25.0)	5 (33.3)	11 (25.6)	13 (24.5)
**Race**							
** Asian**	2 (20.0)	0	0	0	0	0	2 (3.8)
** Black**	0	1(6.7)	0	0	0	1 (2.3)	1 (1.9)
** White**	8 (80.0)	13 (86.7)	5 (100)	5 (62.5)	14 (93.3)	37 (86.0)	45 (84.9)
** Other**	0	1 (6.7)	0	3 (37.5)	1 (6.7)	5 (11.6)	5 (9.4)
**ECOG PS** ^a^							
** 0**	3 (30.0)	6 (40.0)	2 (40.0)	1 (12.5)	4 (26.7)	13 (30.2)	16 (30.2)
** 1**	6 (60.0)	7 (46.7)	3 (60.0)	7 (87.5)	10 (66.7)	27 (62.8)	33 (62.3)
** 2**	1 (10.0)	2 (13.3)	0	0	1 (6.7)	3 (7.0)	4 (7.5)
**Primary site of cancer**							
** Colon, left**	5 (50.0)	3 (20.0)	1 (20.0)	2 (25.0)	6 (40.0)	12 (27.9)	17 (32.1)
** Colon, right**	0	2 (13.3)	0	3 (37.5)	1 (6.7)	6 (14.0)	6 (11.3)
** Rectum**	0	3 (20.0)	2 (40.0)	3 (37.5)	6 (40.0)	14 (32.6)	14 (26.4)
**Other or NS**	5 (50.0)	7 (46.7)	2 (40.0)	0	2 (13.4)	11 (25.6)	16 (32.4)
**No. of prior antineoplastic regimens**							
** 1-2**	1 (10.0)	5 (33.3)	1 (20.0)	3 (37.5)	2 (13.3)	11 (25.6)	12 (22.6)
** ≥3**	9 (90.0)	10 (66.7)	4 (80.0)	5 (62.5)	13 (86.7)	32 (74.4)	41 (77.4)

Data are *n* (%) unless indicated otherwise.

^a^ECOG PS: 0 = without restriction; 1 = restricted in physically strenuous activity but ambulatory and able to carry out work of a light or sedentary nature (eg, light housework, office work); 2 = ambulatory and capable of all self-care but unable to carry out any work activities.

Abbreviations: EGFRi: epidermal growth factor receptor inhibitor; EGFRi-naïve: naïve to epidermal growth factor receptor inhibitor (both monoclonal antibody and tyrosine kinase); ECOG: Eastern Cooperative Oncology Group; NS: not specified; PS: performance status; WT: wild type.

### Maximum Tolerated Dose/Recommended Phase II Dose

No DLTs were reported during phase Ib of the study. The MTD/RP2D was determined to be binimetinib 45 mg BID + panitumumab 6 mg/kg Q2W.

### Efficacy

In phase Ib, no patients had a response to combination therapy with binimetinib + panitumumab; 7 patients had stable disease for a DCR of 70% (95%CI, 34.8-93.3). In phase II, one patient in the *RAS* WT EGFRi-pretreated group had a confirmed PR for an ORR of 6.7% (95%CI, 0.2-31.9) within the group and an ORR for phase II of 2.3% (95%CI, 0.1-12.3). The DOR for this patient was 5.3 months. In addition to the confirmed PR in the *RAS* WT EGFRi-pretreated group, 5 patients had stable disease for a within group DCR of 40% (95%CI, 16.3-67.7). While no confirmed complete responses were observed, 12 of 43 patients had stable disease, for a phase II DCR of 30.2% (95%CI, 17.2-46.1; Table 2). In the *RAS* WT EGFRi-naïve group, one patient had an unconfirmed PR.

**Table 2.** Summary of confirmed response per local assessment.

	Phase Ib (*n* = 10)	Phase II					All patients (*N *= 53)
		*RAS* MUT, EGFRi-naïve (*n* = 15)	Acquired *RAS* MUT, EGFRi pretreated (*n* = 5)	*RAS* WT, EGFRi-naïve (*n* = 8)	*RAS* WT, EGFRi pretreated(*n* = 15)	All phase II (*n *= 43)	
**Best response, *n* (%)**							
** CR**	0	0	0	0	0	0	0
** PR**	0	0	0	0	1 (6.7)	1 (2.3)	1 (1.9)
** SD**	7 (70.0)	2 (13.3)	2 (40.0)	3 (37.5)	5 (33.3)	12 (27.9)	19 (35.8)
** PD**	3 (30.0)	5 (33.3)	3 (40.0)	2 (25.0)	4 (26.7)	14 (32.6)	17 (32.1)
** Unknown**	0	8 (53.3)	0	3 (37.5)	5 (33.3)	16 (37.2)	16 (30.2)
**ORR (CR + PR), *n* (%)**	0	0	0	0	1 (6.7)	1 (2.3)	1 (1.9)
** 95%CI**	NE	NE	NE	NE	0.2-31.9	0.1-12.3	(0.0-10.1)
**DCR (CR + PR + SD), *n* (%)**	7 (70.0)	2 (13.3)	2 (40.0)	3 (37.5)	6 (40.0)	13 (30.2)	20 (37.7)
** 95%CI**	34.8-93.3	1.7-40.5	5.3-85.3	8.5-75.5	16.3-67.7	17.2-46.1	24.8-52.1

Abbreviations: CI: confidence interval; CR: complete response; DCR: disease control rate; EGFRi: epidermal growth factor receptor inhibitor; NE: not estimable; ORR: overall response rate; PD: progressive disease; PR: partial response; *RAS*: rat sarcoma viral oncogene homolog; SD: stable disease; WT: wild type.

**Figure 2.**
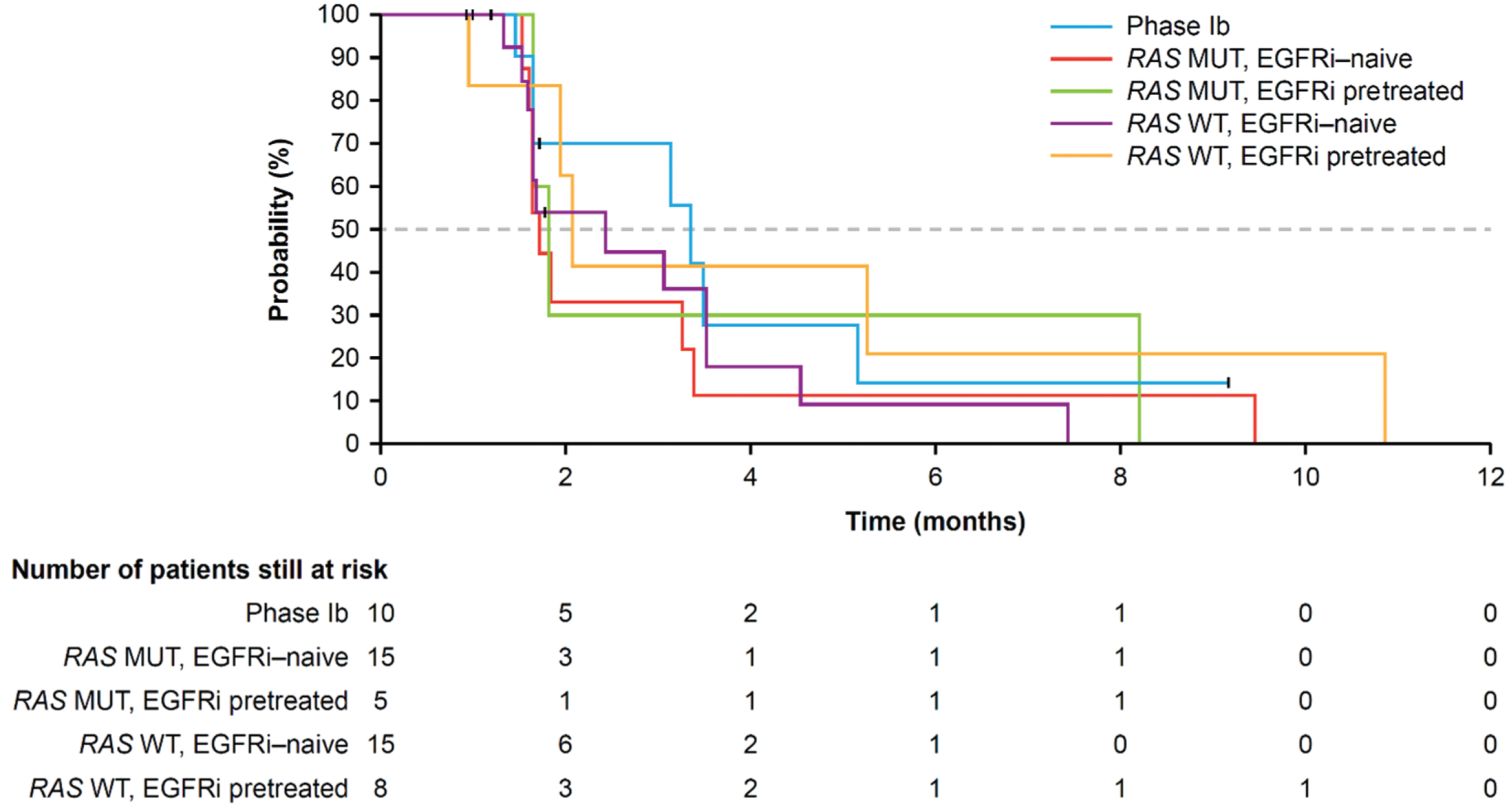
PFS in months, per local assessment. Median PFS: phase Ib; 3.4 months (95%CI, 1.4-5.2). Phase II; all patients: 1.8 months (95%CI, 1.6-3.3); *RAS* MUT, EGFRi-naïve: 1.7 months (95%CI, 1.5-3.4); *RAS* MUT, EGRFi pretreated: 1.8 months (95%CI, 1.6-8.2); *RAS* WT, EGFRi-naïve: 2.1 months (95%CI, 1.0-10.8); *RAS* WT, EGRFi pretreated, 2.4 months (95%CI: 1.6-3.5). Abbreviations: EGFRi: epidermal growth factor receptor inhibitor; MUT: mutation; PFS: progression-free survival; *RAS*: rat sarcoma viral oncogene homolog; WT: wild type.

**Figure 3.**
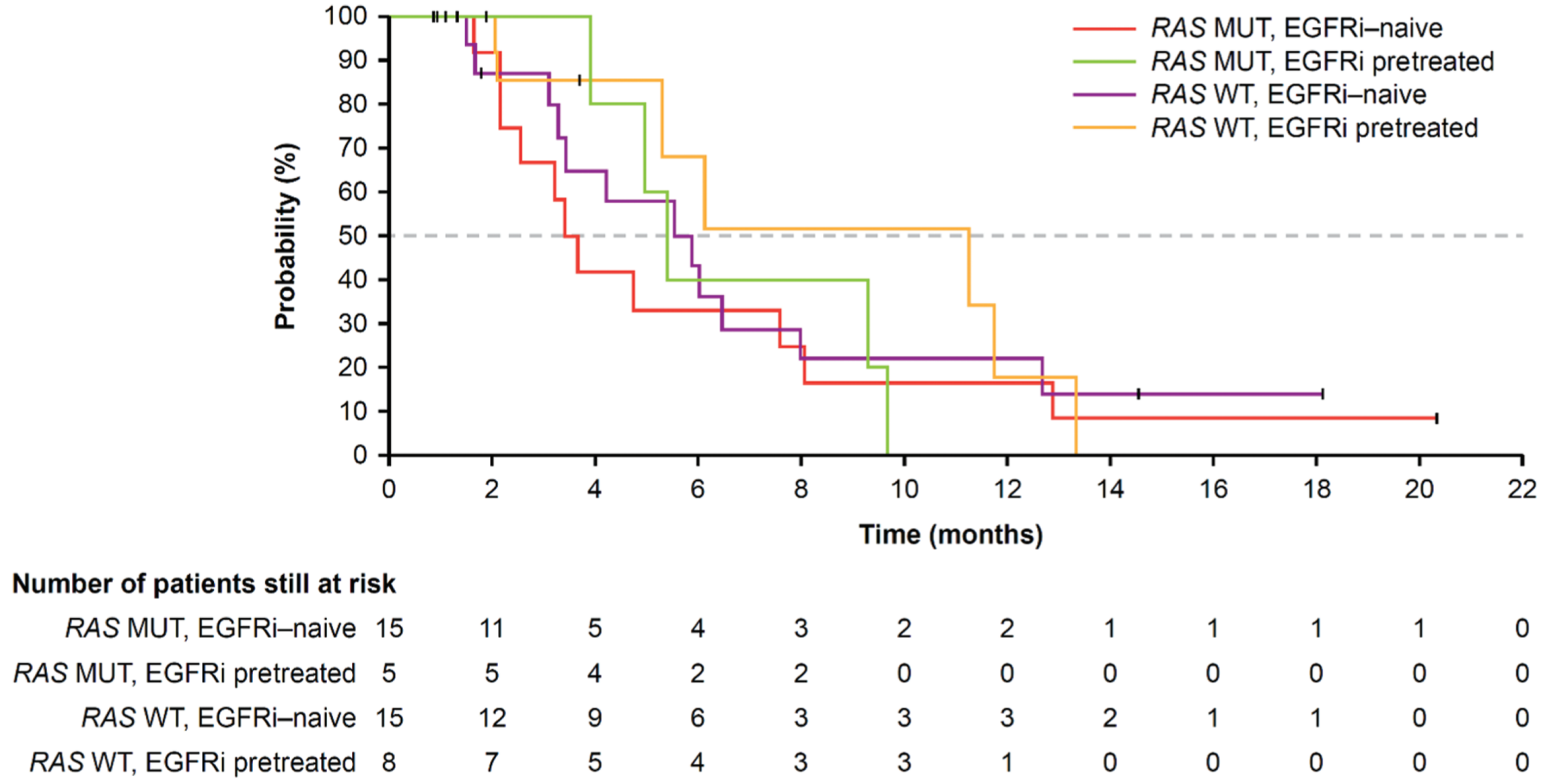
OS in months. Median OS: phase II; all patients: 5.5 months (95%CI, 3.6-7.6); *RAS* MUT, EGFRi-naïve: 3.5 months (95%CI, 2.1-8.0); *RAS* MUT, EGRFi pretreated: 5.5 months (95%CI, 3.9-9.6); *RAS* WT, EGFRi-naïve: 11.2 months (95%CI, 2.1-13.3); *RAS* WT, EGRFi pretreated, 5.8 months (95%CI: 3.1-8.0). Abbreviations: EGFRi: epidermal growth factor receptor inhibitor; MUT: mutation; OS: overall survival; *RAS*: rat sarcoma viral oncogene homolog; WT: wild type.

In phase II, the DCR was lowest in the *RAS* MUT, EGFRi-naïve group (13.3%) and was between 37.5% and 40.0% across the other subgroups (Table 2). Across all patients in phase II, median PFS was 1.8 months (95%CI, 1.6-3.3; Fig. 2), and median OS was 5.5 months (95%CI, 3.6-7.6; Fig. 3). Given the limited clinical activity of binimetinib + panitumumab across subgroups, the study was closed early.

### Safety

The median duration of exposure to binimetinib + panitumumab combination therapy was 9.1 (range 5.3-40.0) weeks in phase Ib and 6.1 (range 1.0-48.0) weeks in phase II of the study (*RAS* MUT, EGFR-naïve: 5.6 weeks; *RAS* MUT, EGRFi pretreated: 8.1 weeks; *RAS* WT, EGFR-naïve: 7.0 weeks; *RAS* WT, EGRFi pretreated: 6.0 weeks).

Across both study phases, all patients experienced ≥1 AE, regardless of study drug relationship; the most common AEs were diarrhea (71.7%), vomiting (52.8%), nausea (50.9%), fatigue (49.1%), dermatitis acneiform (43.4%), and rash (41.5%) (Table 3). In phase Ib, the most commonly reported AEs were fatigue (90.0%), dermatitis acneiform (80.0%), diarrhea (70.0%), dry skin (60.0%), hypomagnesemia (60.0%), nausea (50.0%), increased blood creatinine phosphokinase (50.0%), maculopapular rash (50.0%), and decreased appetite (50.0%). In phase II, the most common AEs were diarrhea (72.1%), vomiting (55.8%), nausea (51.2%), and rash (46.5%). In the full study population, for both rash and diarrhea specifically, the severity was most often grade 1/2 with no grade 4 events reported.

**Table 3.** AEs, regardless of study drug relationship, and select laboratory abnormalities occurring in ≥20% of patients across treatment groups.

	Phase Ib (*n *= 10)	Phase II (*n *= 43)	All patients (*N* = 53)
AEs			
Diarrhea	7 (70.0)	31 (72.1)	38 (71.7)
Vomiting	4 (40.0)	24 (55.8)	28 (52.8)
Nausea	5 (50.0)	22 (51.2)	27 (50.9)
Fatigue	9 (90.0)	17 (39.5)	26 (49.1)
Dermatitis acneiform	8 (80.0)	15 (34.9)	23 (43.4)
Rash	2 (20.0)	20 (46.5)	22 (41.5)
Blood CPK increased	5 (50.0)	12 (27.9)	17 (32.1)
Dry skin	6 (60.0)	11 (25.6)	17 (32.1)
Abdominal pain	1 (10.0)	13 (30.2)	14 (26.4)
Rash, maculopapular	5 (50.0)	8 (18.6)	13 (24.5)
Stomatitis	3 (30.0)	10 (23.3)	13 (24.5)
Constipation	2 (20.0)	10 (23.3)	12 (22.6)
Hypomagnesemia	6 (60.0)	6 (14.0)	12 (22.6)
Decreased appetite	5 (50.0)	6 (14.0)	11 (20.8)
Pyrexia	2 (20.0)	9 (20.9)	11 (20.8)
AESI			
Dermatologic events (rash)	10 (100.0)	38 (88.4)	48 (90.6)
Gastrointestinal events	8 (80.0)	39 (90.7)	47 (88.7)
Fatigue/asthenia	9 (90.0)	24 (55.8)	33 (62.3)
Dermatologic events (skin except rash)	8 (80.0)	16 (37.2)	24 (45.3)
Myopathy/rhabdomyolysis	7 (70.0)	15 (34.9)	22 (41.5)
Retinal events	5 (50.0)	13 (30.2)	18 (34.0)
Edema events	4 (40.0)	10 (23.3)	14 (26.4)
Liver events	0	11 (25.6)	11 (20.8)
Other eye events	3 (30.0)	7 (16.3)	10 (18.9)
Cardiac events	1 (10.0)	6 (14.0)	7 (13.2)
Hemorrhage events	4 (40.0)	3 (7.0)	7 (13.2)
Hypertension events	2 (20.0)	3 (7.0)	5 (9.4)
Thrombotic/embolic events	1 (10.0)	3 (7.0)	4 (7.5)
QTc prolongation	0	1 (2.3)	1 (1.9)

Data are *n* (%).

Abbreviations: AE: adverse event; AESI: adverse event of special interest; CPK: creatine phosphokinase.

In phase Ib, 8 patients (80.0%) reported ≥1 grade 3/4 AE, and 3 patients (30.0%) had serious AEs (SAEs; all grade 3/4). In phase II, 37 patients (86.0%) experienced grade 3/4 AEs and 22 (51.2%) had SAEs, including 17 patients (39.5%) who had grade 3/4 SAEs. Overall, the most common SAEs (>1 patient) included diarrhea (*n* = 4), abdominal pain (*n* = 3), small intestinal obstruction (*n* = 3), pneumonia (*n* = 2), and vomiting (*n* = 2). In phase Ib, 2 patients discontinued study treatment due to AEs, and 9 patients had AEs that led to temporary drug interruption or dose reduction. In phase II, 15 patients (34.9%) discontinued study treatment due to AEs, and 38 patients (88.4%) had AEs that resulted in temporary drug interruption or dose reduction. Across both phases, a total of 7 patients (13.2%) died while receiving treatment or within 30 days of the last dose of study treatment. In phase Ib, 2 patients died due to disease progression. In phase II, 4 patients died due to disease progression and one due to presumed treatment-related hypoxia.

All patients experienced ≥1 AESI, most frequently dermatologic rash (90.6%), gastrointestinal events (88.7%), and fatigue/asthenia (62.3%). During phase Ib, 6 patients had a grade 3/4 AESI; in phase II, 29 patients experienced a grade 3/4 AESI.

## Discussion

This phase Ib/II study aimed to determine the MTD and/or RP2D of the MEKi binimetinib given in combination with the EGFRi panitumumab and to investigate the efficacy and safety of the combination treatment in patients with mCRC. While there were no DLTs at the full dose of both agents, the combination was difficult to tolerate. There was limited clinical activity, and consequently, study enrollment was closed, and disease progression/survival follow-up were discontinued. The 30-day safety follow-up was not changed.

The MTD/RP2D of the combination of binimetinib + panitumumab was determined to be binimetinib 45 mg BID + panitumumab 6 mg/kg Q2W. The treatment demonstrated limited clinical activity in adult patients with either *RAS* MUT or *RAS* WT mCRC. During phase II, only one confirmed PR was recorded for a patient with *RAS* WT mCRC, previously treated with an EGFRi. No confirmed treatment responses were reported in the *RAS* WT, EGFRi-naïve group. When administered at the MTD/RP2D, the treatment combination was poorly tolerated, and the majority of patients in phase II (86.0%) experienced a grade 3/4 AE, with over half of the patients reporting an SAE. Over one-third of the patients discontinued treatment as a result of AEs, and most patients experienced a treatment interruption or dose reduction as a consequence of tolerability issues. It is plausible that the overlapping toxicity profiles of binimetinib and panitumumab^16,19,20^ limited the feasibility of their coadministration, thereby compromising clinical efficacy. Prior studies of combined EGFR and MEK inhibition with panitumumab + trametinib, respectively, suggest that this regimen is highly toxic, with toxicities being primarily dermatologic in nature (dermatitis acneiform).^20,21^ Current trials evaluating MEK inhibitors with broader inhibitors of upstream signaling, including drugs targeting SHP2 and SOS, are ongoing and will determine the safety and efficacy of these combinations.

In this study, patients were enrolled irrespective of primary tumor site. Patients with right-sided colon cancer primary sites are less likely to respond to EGFRi.^22^ The inclusion of these patients, who made up over one-third of the *RAS* WT, EGFRi-naïve group, may have contributed to the low response rate seen in this group.

The reversibility of acquired resistance to EGFRi upon withdrawal of treatment, and the subsequent restoration of drug sensitivity, has been documented in mCRC.^23,24^ The potential benefit of retreatment with EGFRi therapy, after an EGFRi-free interval, expands therapeutic options for patients who initially responded to EGFRi treatment. Our data show similar rates of disease control in patients with *RAS* WT mCRC (irrespective of prior EGFRi exposure) and in those in the acquired *RAS* MUT, EGFRi pretreated subgroup. In addition, in this trial, a RECIST PR was achieved in a patient with EGFRi pretreated *RAS* WT mCRC. For these reasons, retreatment with EGFRi therapy following initial challenge may be beneficial for some patients.

The clinical development of binimetinib in combination with other therapeutic agents for patients with mCRC is ongoing. In the open-label, phase III BEACON study, the combination of encorafenib + cetuximab + binimetinib resulted in a significantly higher OS and ORR than standard therapy in patients with *BRAF* V600E-mutant mCRC.^18,25^ The combination of binimetinib + encorafenib + cetuximab was designed for maximum inhibition of the MAPK pathway to avoid EGFR-mediated adaptive feedback mechanisms that reactivate the pathway.^26^ We note that the presence of encorafenib in this triplet combination likely offsets toxicity from combined MEK and EGFR inhibitors. Alternatively, fewer patients were treated with this combination. The triplet combination was well tolerated in the BEACON study, with a similar rate of treatment discontinuation in the doublet and triplet arms and delayed deterioration of quality of life for patients participating in the doublet and triplet arms.^27,28^

The similar rates of disease control between patient subgroups observed in the current study, regardless of prior EGFRi exposure, may help to further delineate the impact of *RAS* mutations on antitumor activity through MAPK pathway inhibition.

Limitations of this study include the early closure of study enrollment due to a lack of clinical activity and the small number of patients enrolled into phase II, particularly in the acquired *RAS* MUT, EGFRi pretreated and *RAS* WT, EGFRi-naïve subgroups. In addition, *BRAF* V600E mutations status was not available for all patients. Together, these limitations further restrict the generalizability of these findings.

In conclusion, combination treatment with binimetinib + panitumumab exhibited limited clinical activity in adult patients with *RAS* MUT or *RAS* WT mCRC, regardless of EGFRi treatment history. This combination did not improve activity of EGFRi compared with historical data for EGFRi alone and did not overcome EGFRi resistance in *RAS* MUT mCRC. The safety profile of the combination further limits its feasibility as a clinical treatment approach in patients with mCRC.

## Data Availability

Upon request, and subject to review, Pfizer will provide the data supporting the findings of this study. Subject to certain criteria, conditions, and exceptions, Pfizer may also provide access to the related individual deidentified participant data. See https://www.pfizer.com/science/clinical-trials/data-and-results for more information.
